# Interictal epileptiform discharges in Alzheimer’s disease: prevalence, relevance, and controversies

**DOI:** 10.3389/fneur.2023.1261136

**Published:** 2023-09-21

**Authors:** Hernan Nicolas Lemus, Rani A. Sarkis

**Affiliations:** Department of Neurology, Brigham and Women’s Hospital, Boston, MA, United States

**Keywords:** Alzheimer’s disease, dementia, electroencephalography, interictal discharges, epileptogenesis

## Abstract

Alzheimer’s disease (AD) is the most common type of dementia and remains an incurable, progressive disease with limited disease-modifying interventions available. In patients with AD, interictal epileptiform discharges (IEDs) have been identified in up to 54% of combined cohorts of mild cognitive impairment (MCI) or mild dementia and are a marker of a more aggressive disease course. Studies assessing the role of IEDs in AD are limited by the lack of standardization in the definition of IEDs or the different neurophysiologic techniques used to capture them. IEDs are an appealing treatment target given the availability of EEG and anti-seizure medications. There remains uncertainty regarding when to treat IEDs, the optimal drug and dose for treatment, and the impact of treatment on disease course. This review covers the state of knowledge of the field of IEDs in AD, and the steps needed to move the field forward.

## 1. Introduction

Alzheimer’s disease (AD) is the most common type of dementia with devastating effects on cognition in the setting of disrupted synaptic homeostasis, neuronal loss, and impaired neuronal network integrity (1, 2). Clinical studies have suggested a link between AD and epilepsy, given the higher rates of clinical and subclinical seizures in patients with AD (3) and a more aggressive phenotype (early onset and rapid progression) when both disorders are present, or when there is evidence of interictal epileptiform discharges (IEDs) on EEG even in the absence of clinical seizures (4). Seizures can also be one of the first presenting symptoms of AD (4, 5), and an “epileptic variant” of AD is gaining more recognition (6). The accumulation of amyloid-β (a pathogenic hallmark of AD) leads to inhibitory interneuron dysfunction creating a state of network hypersynchrony manifesting as IEDs, clinical and subclinical seizures (7). This raises the appealing prospect of trying to modify the disease course by addressing hypersynchrony with antiseizure medications (ASM).

Electroencephalography (EEG) has proven to be the most accessible and cost-efficient tool to identify epileptiform abnormalities in patients with mild cognitive impairment (MCI) or Alzheimer’s dementia (4, 5). Albeit there may be substantial variability in the interpretation and reporting of the data (8). There is a need for clinicians to understand the EEG findings in patients with AD, its role in the pathogenesis and progression of the disease, and when and whether certain findings should be treated. This review will highlight published data regarding IEDs in AD, and discuss study limitations, and controversies regarding treatment.

## 2. Illustrative cases

We present 4 cases with different EEG findings in the setting of non-lesional MRIs and highlight the range of abnormalities that a clinician could face, and the challenge with regards to deciding when to initiate treatment.

**Figure 1:**
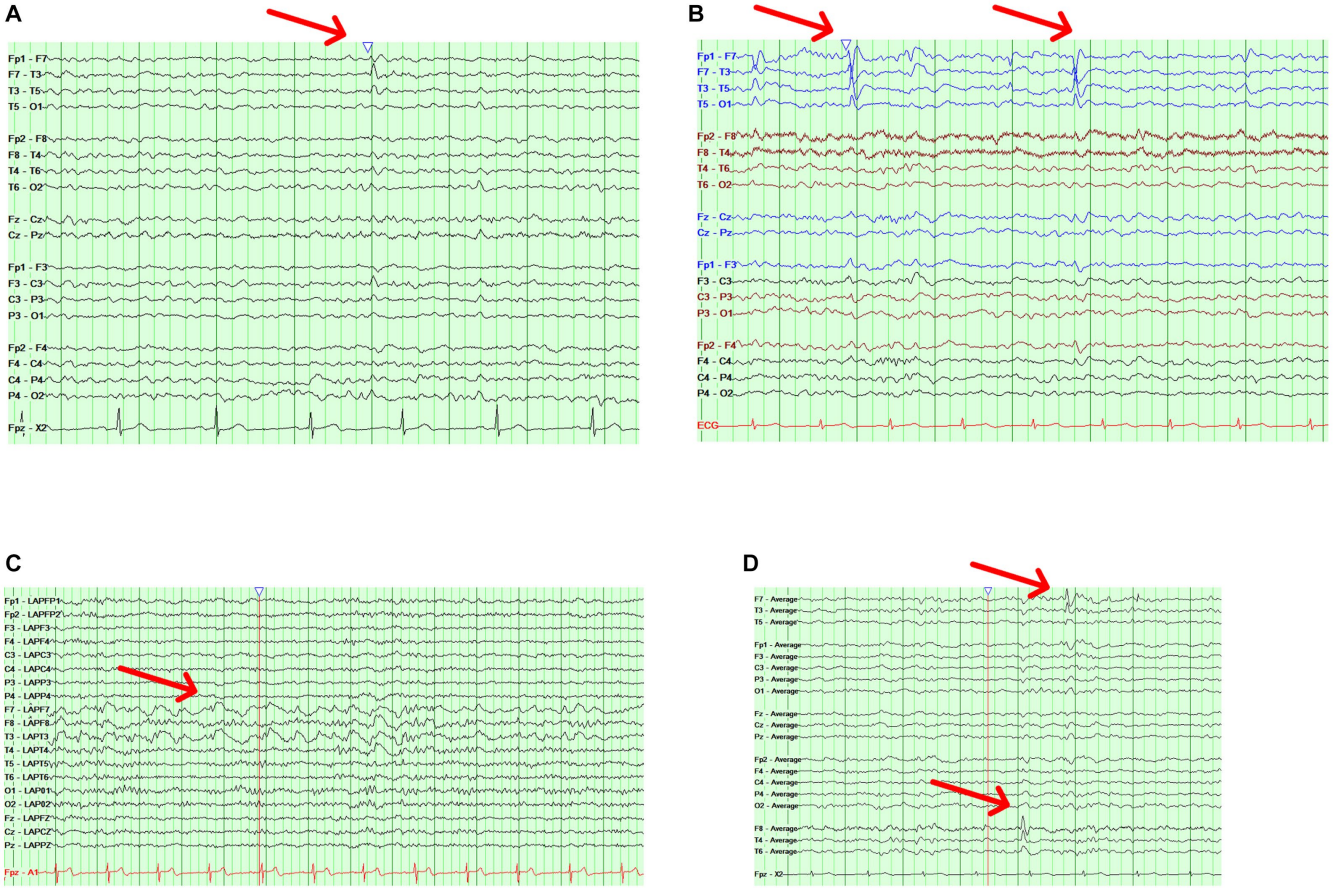
Illustrative cases. **(A)** Case 1 with an ambulatory EEG showing a bipolar longitudinal montage with a left temporal sharp wave (red arrow) during stage N2 sleep. **(B)** Case 2 with an ambulatory EEG showing a bipolar longitudinal montage with occasional periodic left temporal sharp waves (red arrow) during stage N2 sleep. **(C)** Case 3 with an ambulatory EEG showing a Laplacian montage with runs of left temporal rhythmic delta activity (red arrow) lasting up to 10 s. **(D)** Case 4 with an ambulatory EEG showing an average referential montage with bitemporal independent frequent spike and slow wave discharges (red arrows).

Case 1 is a 75-year-old male with a history of mild cognitive impairment and no history of spells concerning for seizures. He had a routine EEG revealing “sharp transients in sleep.” An ambulatory EEG showed an isolated left anterior temporal sharp wave in N2 sleep (Figure 1A). Case 2 is a 60-year-old female with a strong family history of AD who presented to the clinic with short-term memory complaints. An ambulatory EEG was obtained revealing occasional periodic left temporal sharp waves in N2 sleep (Figure 1B). Case 3 is an 85-year-old female with a history of mild AD dementia and fluctuating mentation. An ambulatory EEG showed runs of left temporal rhythmic delta activity lasting up to 10 s limited to wakefulness (Figure 1C). Case 4 is a 70-year-old male with a history of mild cognitive impairment and an isolated generalized tonic–clonic seizure; he was maintained on levetiracetam monotherapy. A follow-up ambulatory EEG showed bitemporal independent frequent spike and slow wave discharges in sleep occurring at a frequency of 1/min (Figure 1D).

## 3. EEG findings in AD

Earlier studies in patients with AD suggested that slowing of the occipital peak frequency correlated with the progression of the disease (9). Unfortunately, larger cohort studies failed to confirm this finding (10). Focal slowing on ambulatory EEG is a common finding in older adults in general with a prevalence of up to 63% on ambulatory EEG (11). Focal slowing on a routine EEG is relevant because it is one of the predictors of finding an IED on an ambulatory EEG (11). In the AD literature, the rates of focal slowing range between 44–47% in MCI and mild AD (12), while generalized slowing of the background is seen in 13–30% (3, 12).

More recent studies have highlighted interictal epileptiform discharges (IEDs) as a more relevant electrographic finding in patients with AD or MCI (Table 1); as also previously reviewed by Csernus and colleagues (20). Most of the study findings are limited by the lack of standardization in the definition of IEDs or the variability in the type of study used to capture them (routine vs. long-term vs. ambulatory EEG). An interictal epileptiform abnormality should be defined by at least 4 out of 6 criteria recommended by the international federation for clinical neurophysiology to avoid misinterpretation of normal variants on EEG (19). Common normal variants in older adults that can be easily misinterpreted as pathologic include small sharp spikes, wicket rhythms, and wicket spikes (8). Yet, even when these criteria are applied, there may be substantial interrater variability; the inter-rater reliability regarding IEDs is fair at best even among experts (21). This is problematic because AD patients without a history of seizures tend to have only a limited number of discharges on an ambulatory EEG (12) and can be easily misclassified as having IEDs, as shown in the illustrative cases (Figure 1) where the decision to label an EEG as epileptiform or not rests on an isolated discharge.

**Table 1:** Characteristics of IEDs in AD.

Modality of EEG used/ EEG findings	Incidence; Frequency of IEDs on EEG	AD biomarkers available	Most common EEG scalp localization of IEDs	Most common EEG state for IEDs	Spike detection software used	Inter-rate agreement among EEG raters for IEDs	Number of patients studied	Reference
Unclear duration of EEG/ Spike or sharp wave discharges	15 patients (38%); N/A	N/A	Uni or bitemporal	N/A	N/A	N/A	39 patients with dementia from a registry (74% undergo an EEG)	(13)
REEG/ IEDs as per IFCN criteria#	42 patients (3%); N/A	N/A	Temporal	N/A	N/A	1 of 3 board-certified clinical neurophysiologists	1,674 patients attending a memory clinic	(14)
LTM and REEG/ Sharp waves, Generalized ED, Focal and diffuse slowing	aMCI or AD evaluated for sz vs. no history of sz: 62% vs. 6%; N/A	6/54 *2 had autopsy confirmed AD	Temporal (Left)	N/A	N/A	N/A	aMCI + epilepsy: 12 AD plus epilepsy: 35 AD plus IEDs: 7	(4)
LTM and REEG/ Epileptiform abnormalities, focal and generalized slowing	36% with epileptiform abnormalities; N/A	N/A	Frontal or temporal	N/A	N/A	N/A, retrospective study	77 patients (88% with possible/ probable/definite AD)	(5)
REEG/ epileptiform discharges (sharp waves or spikes)	23.1% with epileptiform abnormalities; N/A	13/13	Temporal (Left>Right)	N/A	N/A	N/A	13 patients with AD (MCI) and epilepsy	(6)
LTM/ Epileptiform activity+	21.2% vs. 0%; 0.03 to 5.18 per hour	25/33	Temporal (Left)	Sleep (Stage 2)	SpikeDensityV101 Calculation Engine in Persyst 11 EEG software	Two experienced epileptologists	33 patients with AD; 19 HC	(3)
AEEG/ Epileptiform activity+	AD vs. HC: 54% vs. 25%; 0.29–6.68 spikes/h	N/A	Temporal (left)	Sleep (Stage 3)	Micromed System PLUS98, Compumedics NeuroScan Curry	Two independent raters	52 patients with AD 20 HC	(15)
Overnight EEG + PSG/ Epileptiform activity*	Probable AD vs. MCI vs. Controls: 6.38% vs. 11.63% vs. 4.54%; N/A	N/A	N/A	N/A	(RembrandtSleep-View, Medcare)	Two trained neurophysiologists	Probable AD: 47 MCI: 43 Controls: 44	(16)
AEEG (24 h)/ Epileptiform discharges	AD-no epilepsy vs. AD-epilepsy vs. controls: 22% vs. 53.3% vs. 4.7%; 0 to 0.41/h in AD-no epilepsy 0 to 53.3/h in AD-epilepsy	18/56 1 autopsy confirmed	Temporal (Left>Right)	Sleep (Stage 2)	Manual; Matlab for revision	Two trained epileptologists screen IEDs followed by a consensus among 9 epileptologists	AD-no epilepsy: 41 (27 MCI) AD-epilepsy: 15 (10 MCI) Controls: 43	(12)
LTM-EEG (24 h)/ Epileptiform activity+	AD vs. healthy controls: 54% vs. 25%; 0.29–6.68 per hour	N/A	Temporal (Left)	Sleep (stages 2 and 3)	Micromed System PLUS98, Compumedics NeuroScan Curry	Two independent raters	AD: 52; HC: 20	(17)
Ear-EEG^ and 30-min REEG/ ED as per IFCN criteria#	AD vs. HC: 75% vs. 46.7%; mean: 3.03 spikes per 24 h	20/24	Set up was limited to Ear-EEG.	At night (64.8%)	N/A	Two experienced clinical neurophysiologists	AD: 25 HC: 15	(18)

LTM-EEG, long-term EEG; REEG, routine EEG; AEEG, ambulatory EEG; ED, epileptiform discharges; AD, Alzheimer’s disease; aMCI, amnestic mild cognitive impairment; Sz, seizures; IEDs, interictal epileptiform discharges; N/A, not available for review; HC, healthy controls. +, Defined as paroxysmal sharp waveforms 20–200 ms, clearly distinct from ongoing background activity, with an associated subsequent slow wave. *, Defined as paroxysmal EEG sharp grapho elements that disrupted background activity lasting from 20 to 200 ms, with an abrupt change in polarity. #, as defined by the IFCN (19); notably, Ear-EEG cannot identify slow waves as reported by the authors (18). ^ Ear-EEG, defined as electrodes placed inside the ears.

Notably, only one study has used expert consensus to evaluate the frequency of IEDs in patients with AD (12), while others screened with spike detection software followed by a visual review (Table 1). Recent studies have suggested a higher accuracy in the identification of IEDs for an ambulatory EEG when compared to one or two routine EEGs (22). This could be relevant in the AD population since most IEDs are present in stage 2 sleep (Table 1). The sensitivity of an EEG is correlated with the length of the recording, which explains why a 20–30 min routine EEG may miss IEDs (23), and why there was a delay in appreciating the true burden of IEDs in AD. Other markers of hyperexcitability such as focal rhythmic slowing (24) have only been studied in one cohort (12). A *benign* variant, small sharp spikes (SSS), was seen in a subset of patients with AD; some with a high frequency and unilateral predominance (12), suggesting that these features may also indicate underlying irritability given that they represent outliers and also tended to co-occur in EEGs with IEDs. Most of the studies also reported the temporal lobe as the most frequent region for IEDs (Table 1). The temporal-lobe predominance of IEDs could be due to early seeding by amyloid plaques and hyperphosphorylated tau in the limbic system (25).

It must be kept in mind that surface EEG as a neurophysiologic tool has several limitations including its limited ability to detect deep IEDs such as those located in the hippocampus, or IEDs with a tangential dipole (26). This limitation was highlighted in a study in 2016, where 21% out of 42% of the subjects had MEG-only IEDs with no IEDs noted on EEG (3). Similarly, in a case series of 2 subjects with early onset AD with surface EEG and invasive foramen ovale electrodes, 90–100% of the IEDs noted on the invasive electrodes did not have a surface EEG correlate (27).

As illustrated in Table 1 the subjects examined per study with prolonged EEGs have been limited to date with cohorts often including both MCI and mild dementia patients lumped together. We need more studies to explore whether IEDs vary in prevalence depending on disease stage.

## 4. IEDs and cognition in the epilepsy literature

The association of IEDs on cognition and whether they should be a treatment target has been a matter of debate among epileptologists (28). Transient cognitive impairment secondary to IEDs gained recognition with the advent of computerized testing paradigms. Earlier studies showed that around 50% of subjects with epilepsy exhibited transient impairment coinciding with the occurrence of an IED, and there was a laterality effect with left-sided discharges affecting verbal tasks while right-sided IEDs affecting visual ones (29, 30). The dysfunction was specifically attributed to the after-going slow wave following the discharge (31). IEDs can also affect cognition when occurring in sleep by affecting sleep-dependent memory consolidation. Sleep is essential in transitioning memories from being hippocampal-dependent into more consolidated memories in widespread cortical networks (32). This process is dependent on NREM sleep with slow oscillations and sleep spindles playing a pivotal role (32). In older adults with epilepsy, the frequency of scalp-detected IEDs in NREM sleep was found to negatively correlate with 24 h recall on a visual memory task (33).

Moving on from surface EEG-based studies, a similar theme also emerges with invasive EEG studies. Hippocampal IEDs detected on depths electrodes were associated with impaired maintenance and retrieval but not encoding on a short-term memory task (34), while the frequency of IEDs detected during sleep was associated with impaired one-week long-term recall (35). IEDs even outside of the epileptogenic zone have also been associated with impaired cognition (36).

Invasive EEG studies have also shed light on how IEDs can disrupt cognitive processes; one mechanism is through a transient decrease in global functional connectivity (37), while another is through the impairment of spindle generation (38) and the induction of pathologic hippocampal-cortical coupling (39). IEDs may also alter the firing of hippocampal neurons leading to a state of transient cognitive impairment (40, 41).

Other markers of epileptogenicity that can be detected using scalp EEG, and have been described in epilepsy patients, include high frequency oscillations (HFOs) (42). They are currently divided into physiologic and pathologic HFOs. Physiologic HFOs have been shown to play a central role in information retrieval and sleep dependent memory consolidation (43, 44). On the other hand, one of the features of pathologic HFOs is that they tend to coincide with IEDs and occur during the earliest stages of non-REM sleep (45). HFOs pose methodological challenges in their recording and detection (46), thus limiting their widespread clinical use in patients with AD; especially since it is difficult to disentangle pathologic from physiologic HFOs.

## 5. IEDs and cognition in the AD literature

Cross-sectional studies of IEDs in AD show a trend for lower mini-mental status exam (MMSE) scores in those with IEDs (14), although this finding was not seen in a study using prolonged ear-EEG recordings (18). Longitudinal studies of AD patients with IEDs have been limited. In a study of 33 patients with AD, those with IEDs had an accelerated decline in their MMSE score and their executive function composite Z-score (a combination of design fluency, information processing speed, and cognitive control from the Stroop test, digit span backward, modified trails and the California verbal learning test) (3). Of note, not all participants had data on the individual tests, and there was no evidence of a decline in the episodic memory, language, or visuospatial function domains (3). The cohort studied predominantly consisted of patients with early-onset AD and 33% with atypical presentations.

In another study, 28 out of 52 AD patients were noted to have IEDs (17). The authors used the cognitive assessments consisting of a Hungarian version of the Addenbrooke Cognitive Examination (ACE); scored from 0–100 and allowing the extraction of MMSE scores, and analysis of the following cognitive subdomains: orientation, attention, memory, verbal fluency, language, and visuospatial ability (17). When compared to AD patients without IEDs, those with IEDs exhibited a faster decline in ACE scores over 3 years (12.15 points per year vs. 8.17 points per year) and on the MMSE (2.71 points per year vs. 2.22 points per year). The study also found a correlation between IED frequency and the rate of decline in the ACE. In comparison, studies evaluating AD patients with comorbid epilepsy treated with anti-seizure medications (ASMs) did not show a change in MMSE scores over at least a 3-year follow-up (47).

## 6. To treat or not to treat: management of IEDs in AD

Although there is mounting evidence regarding the association between IEDs and impaired cognition and accelerated disease course, there are currently no guidelines to screen for IEDs in AD or to treat IEDs. The goal of the treatment is not seizure prevention because there are no currently anti-epileptogenic medications available. Instead, the aim would be to prevent the possible impact of the IEDs on cognition and memory consolidation. In addition, there is also evidence of neuronal hyperactivity (IEDs being one manifestation of this) causing accelerated neurodegeneration by promoting AD pathology (48). The medication that has garnered the most interest has been levetiracetam. Animal AD mouse models exposed to levetiracetam show IED suppression and improvement in cognition (49). In one of the only randomized trials of the treatment of seizures in AD, levetiracetam (dose range 500-2000 mg) was better tolerated when compared to phenobarbital (dose range 50-100 mg) or lamotrigine (dose range 25-100 mg) and was correlated with improved MMSE scores after 1 year (50). Studies evaluating the IED suppression properties of ASMs in epilepsy also show evidence for lamotrigine and topiramate (51). The downside of treatment is that ASMs in general, as a drug class, are commonly associated with cognitive and fatigue side effects (52). While levetiracetam is associated with prominent neuropsychiatric side effects (53), lamotrigine and other sodium channel blockers are risk factors for falls (54). In addition, benzodiazepines are known to increase the risk of cognitive decline and dementia in the elderly (55).

A recent study trying to tackle the balance between IED suppression and adverse effects of ASMs showed that in children undergoing invasive EEG, reaction time improved with IED suppression (with oxcarbazepine) and worsened with increased IED frequency (56). In this study, levetiracetam did not show a clear benefit (56). In a retrospective analysis of older Japanese patients with IEDs on EEG, treatment with various ASMs improved serial 7 scores and MMSE scores in those with IED suppression (57). The first randomized trial for levetiracetam in AD was published in 2021 (58), and several other trials also exploring levetiracetam are pending. In the trial, 34 patients with AD were treated with levetiracetam at a low dose of 125 mg twice a day vs. placebo and then underwent a washout period and cross-over. Based on overnight EEG and then a 1 h MEG, 13 participants were found to have IEDs. The cognitive battery consisted of the National Institutes of Health Executive Abilities: Measures and Instruments for Neurobehavioral Evaluation and Research (NIH-EXAMINER) which consists of a test measuring executive functions, Stroop color and word test, the Alzheimer’s Disease Assessment Scale—Cognitive Subscale (ADAS-Cog), and a virtual route learning test. There was no improvement on the primary endpoints with the medication, however, a subset analysis of those with IEDs showed that they improved on the virtual route learning test and a subscale of the Stroop test. Notably, there was no evidence of IED suppression with the medications (58).

## 7. How to deal with IEDs in AD patients in the clinical practice

**Figure 2:**
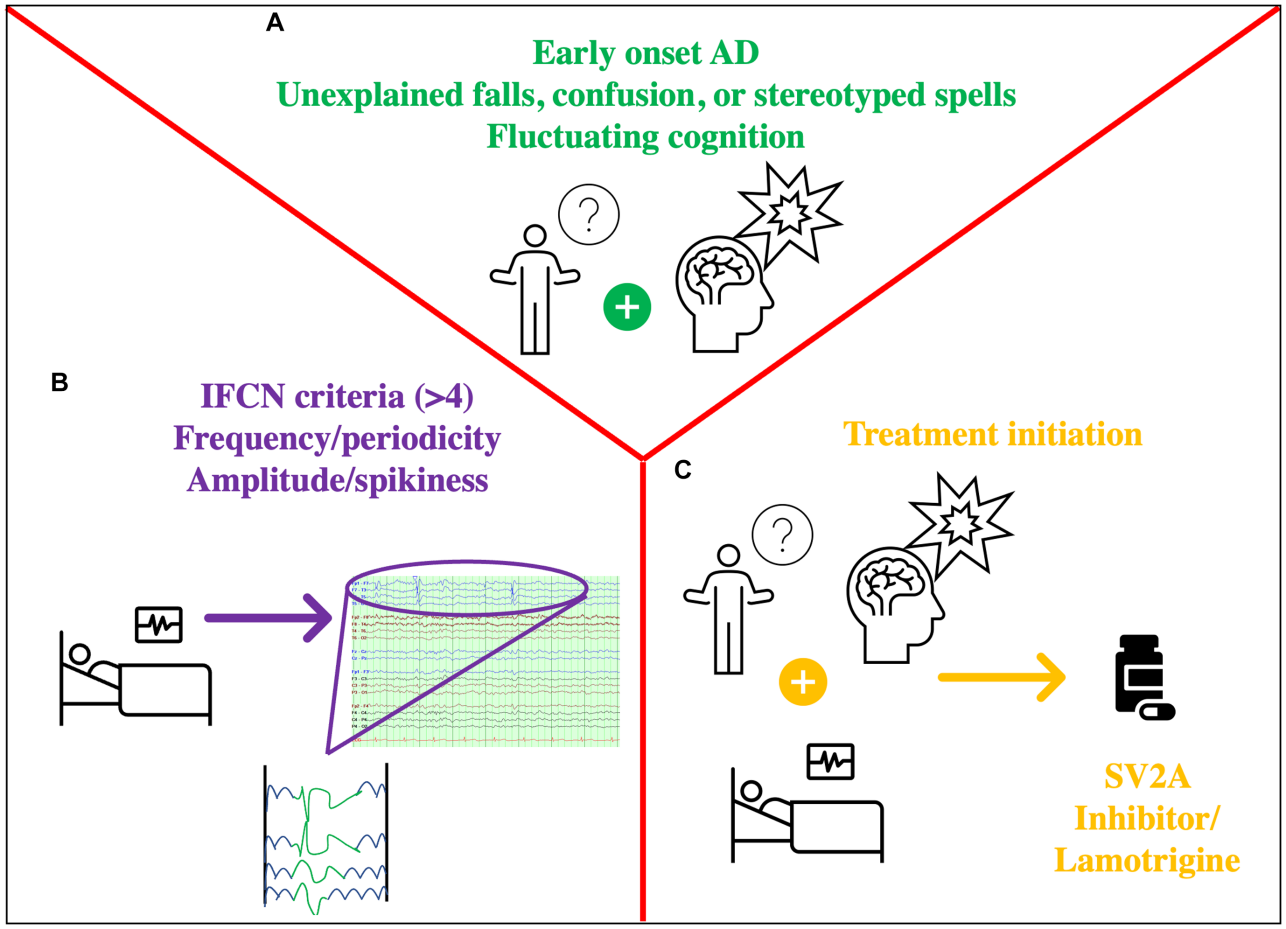
Approach to Hyperexcitability in IED. **(A)** History prompting the need for EEG: fluctuating cognition, stereotyped symptoms, distinct confusional spells, (possibly) early-onset AD **(B)** Concerning EEG features: markers of hyperexcitability such as IEDs with >4 out of 6 criteria of the IFCN, unilateral small sharp spikes, temporal rhythmic delta activity. Assess frequency, periodicity, (possibly) amplitude/spikiness. **(C)** Decision to treat based on A + B: consider an SV2A inhibitor such as levetiracetam/(possibly) brivaracetam or lamotrigine.

Ultimately, the clinician caring for patients with AD is faced with decisions regarding when to order an EEG, how to interpret the data, or when to start an ASM. The other challenge is that diagnosis of epilepsy in an elderly population is challenging, requiring a detailed description of suspected events, consideration of atypical events as seizures (i.e., unexplained falls or brief episodes of confusion), and the need for an expert evaluation (59) (Figure 2). Until we have more evidence from randomized trials that levetiracetam will help AD patients, and more so those with IEDs, routine screening of AD patients with EEG is not recommended. However, one should have a low threshold to screen patients with suspected co-morbid seizures, including those with early onset AD because they are at the highest risk. If an EEG is ordered, it should at least have N2 sleep captured, and that is why 24 h EEGs are preferred over routine EEGs. Interictal discharges as exemplified by the illustrative cases lie along a spectrum, with seizures (clinical and subclinical) occurring at the end of that spectrum and representing the extreme manifestation of network hyperexcitability. Features such as a high IED frequency, periodicity, duration, and perhaps morphological features (spikiness, amplitude) should be considered more concerning and should tip the scale toward treatment (cases 2,3,4). In the absence of more data, isolated and equivocal discharges should not be treated (case 1). When a decision is made to treat, the lowest therapeutic dose should be used to ensure tolerability.

## 8. Conclusion

Network hyperexcitability is a feature of AD, and IEDs are a marker of this phenomenon. They are highly prevalent in AD, are often detected in sleep, and have been linked with deleterious effects on cognition and an accelerated disease course. Limited studies to date show some benefit with treatment, however further evidence is needed to determine whether this should become the standard of care.

## Author contributions

HL: Conceptualization, Writing – original draft, Writing – review & editing. RS: Conceptualization, Writing – original draft, Writing – review & editing.
